# Fleeing Iraq with nothing but a dream

**DOI:** 10.7554/eLife.90402

**Published:** 2023-06-27

**Authors:** Arby Abood

**Affiliations:** 1 Center for Public Health Genomics at The University of Virginia School of Medicine Charlottesville United States

**Keywords:** sparks of change, research culture, discrimination, mental health, resilience, education

## Abstract

Facing the harsh realities of life as a refugee in Jordan and the United States, an ambitious young man holds to a conviction: that he will, one day, get a PhD.

Out in the darkness, a red light on the wing blinks and illuminates a tiny patch of void. My plane ascends into the night sky, leaving Jordan behind; I am bound for the United States. There is anticipation inside my anxiety, a beacon of possibility in the unknown that lies ahead. As a 19-year-old Iraqi refugee, I have been offered a fresh start and maybe, finally, a chance to pursue my dream of becoming a scientist. I knew that there would be challenges ahead; what I had not quite anticipated was that my first real experience of ‘culture shock’ would not take place when I arrived in the United States, but seven years later when I became a Master’s student.

Back when I was 15, my mother and I had left Iraq for Jordan, fleeing the sectarian conflicts that erupted after the 2003 invasion. With that, I left behind my life as a member of a highly educated family, the embodiment of privilege in a country ravaged by unending conflict and poverty. Several wars, years of economic sanctions and increasing religious tensions had severely limited opportunities for even the semblance of a comfortable life in Iraq. Yet I grew up with a fervent passion for education, and a clear goal: to one day become a scientist, and to help to improve the lives of others.

This dream collapsed as I continued my education in Jordan. My mother and I were now refugees with limited resources, and my teachers had readily acknowledged that they were ill-equipped to help students like me face the fierce competition to get into the best universities in the country. In my final year of high school, it became clear anyway that my family would never be able to pay for my education as a foreign student in Jordan. Due to the ongoing violence, going back to Iraq wasn’t an option either.

Stuck in limbo after graduation, I worked as a dishwasher at a local restaurant – illegally, as Iraqi refugees have no official status and are not permitted to work in Jordan. The owner was a Lebanese businessman who liked my work ethic, and he used his connections to help me to apply to a scholarship program which placed Iraqi students in Jordanian universities. I got in, but there were no spots available to study science. I resigned myself to the fact that I would probably never become a scientist: a general studies major was not what I had dreamed of, but it was better than not going to university. Four months later, however, everything changed when I received my refugee visa allowing me to move to the United States.

Within a few weeks of landing in my new country, I had found a job as an overnight cashier at a pharmacy; a month later, I was spending hours poring over books from the library, studying for my first-ever standardised tests so I could join the local community college. Over the next four years, I juggled being a full-time student and a full-time worker, struggling to make ends meet in Northern Virginia, one of the most expensive places to live in the United States. This also meant that I was never able to find time to volunteer to do research. I sometimes felt discouraged, but incredible people who also had escaped their own troubled homelands rallied around me and encouraged me to carry on. After graduating with a 3.93 GPA, I spent two years working as an academic advisor and a lab assistant so I could gain more experience. I began applying for PhD positions… and was rejected everywhere, except for a Master’s program in the largely conservative state of South Carolina. Once again, I felt I had been thrown a lifeline. And so, in the spring of 2015, as the presidential campaigns were picking up speed across the country, I was getting ready to finally start this long-awaited chapter of my life.

My new institution was much less diverse than my alma mater, and I soon realised that life there would be far from easy. As the only Arab American in my department, I often felt alienated. “Good price, just for you”, a professor who was ex-army and had served in the Middle East liked to say, in a thick accent, or ask had I “parked my camel” outside of the lab. For a while, I hoped that their comments and stereotyping stemmed from a place of ignorance, but as the political climate worsened over the next few years, it became clear that blatant hate and racism were commonplace on campus, and that changing their opinion of me would be an uphill battle. In the lab, I was struggling as well: experiments were failing, and the samples I was promised to complete my thesis never materialised. I was told repeatedly that I was not good enough to get a Master’s degree, let alone a PhD.

Little by little, I started to lose sight of my goals, my sense of self and, ultimately, my self-worth. I felt belittled, ostracized, stuck; most of all, I felt helpless. It was all happening again. My Jordanian teachers and classmates had also treated me differently, simply due to my background, and ridiculed me for my desire to become a scientist. Tens of thousands of Iraqis had escaped to Jordan in the aftermath of the 2003 invasion, and we were often harassed and bullied by the local population. Every few months, my mother and I had made the trip to the Iraqi border to renew our visa, and every time, I was reminded that I was not supposed to succeed in this new country. Perhaps I wasn’t supposed to succeed in the United States either. My past trauma finally caught up with me, and I tipped into a deep depression.

In time, I started to explore the concept of emotional intelligence and the benefits of therapy, and I ultimately made peace with myself. I realized that no place is inherently bad, and that the situation was different from when I was a teenager in Jordan. Here, I was an American citizen. I was not stuck. Circumstances beyond my control had driven us to Jordan, but my own decisions had led me to this Master’s program, and it was my responsibility to see it through and find a way out. I decided to carry on with my degree despite the challenges, my love for research acting as a beacon during these trying times. I started to apply to programs back in Virginia, the place where I had once thrived and formed a supportive community. I didn’t have a lot of hope: with no research experience during my undergraduate degree, and no first author publications during my Master’s, I felt I had too many academic shortcomings. Yet, in 2018, I got in. Once again, a glimmer of hope illuminated the void.

Meeting my future PhD advisor during one of my rotations was the event that ultimately changed my path. His research was on a topic I knew relatively little about, but he decided to take a chance on me. In his lab, I finally found a place where I could heal from the scars left by years of trauma. He gave me the time and resources to grow, even paying for educational resources, classes and lodging so I could gain more experience and become competitive on the job market.

He also supported me as I delved deeper into therapy, which was not easy at first. I had to overcome the internalized stigma associated with mental struggles, and it took time for me to understand that seeking help is a sign of strength and self-awareness, that it’s okay to feel vulnerable and to lean on others for support when the burden becomes too heavy. This work allowed me to unearth and confront deep-seated biases and discomforts, often stemming from my experiences. I learned how to better identify, evaluate, control, and express my emotions, which was positive both personally and professionally. More importantly, in therapy I could finally unpack the experiences that had caused me pain, and develop healthier ways to interact with the world around me. This process of introspection and healing didn't just help me survive my struggles – it helped me grow from them and provided me with tools to anticipate and manage future stressors. Through this, I became a more compassionate, understanding, and patient person, and a better scientist too. I can only encourage others to break the silence around mental health and to seek support when needed.

In the end, these experiences helped me realise that my academic ‘shortcomings’ were not personal failures: rather, I had simply been unable to fulfil all the expectations put forward by some members of the academic ivory tower. I hope that, going forward, more graduate programs will act on their promise to consider the personal circumstances of the applicants, and take experiences outside of research into account. Overall, I hope that they will work to improve faculty-trainee relationships, and to address the varied types of harassment faced by trainees.

Two months ago, I defended my thesis and obtained my PhD. I am now a senior scientist in bioinformatics at a wonderful company. I spent decades dreaming about getting a PhD, but I never truly reflected on the true meaning of becoming a scientist. Now I have realized that, for me, it goes beyond the prestige of the degree, or the intricacies of research; it is about finding yourself and the people who matter along the way. When I look back on the challenging periods of my life, I often think of this poem by Tupac Shakur: “*Did you hear about the rose that grew from a crack in the concrete?/Proving nature’s laws wrong, it learned to walk without having feet/Funny it seems, but by keeping its dreams, it learned to breathe fresh air/ Long live the rose that grew from concrete when no one else ever cared*.” Like the rose, keeping my dream allowed me to regain the focus of who I was and what I could do, even when some were trying to prove me otherwise.

## Share your experiences

This article is a Sparks of Change column, where people around the world share moments that illustrate how research culture is or should be changing. Have an interesting story to tell? See what we’re looking for and the best ways to get in touch here.

